# A qualitative study on factors influencing the situational and contextual motivation of medical specialists

**DOI:** 10.5116/ijme.5e88.b9ff

**Published:** 2020-06-19

**Authors:** Stéphanie M.E. van der Burgt, Klaas Nauta, Gerda croiset, Rashmi A. Kusurkar, Saskia M. Peerdeman

**Affiliations:** 1 Amsterdam UMC, Vrije Universiteit Amsterdam, Faculty of Medicine, Research in Education, Amsterdam, the Netherlands; 2 Department of Psychiatry, Amsterdam UMC, location VUmc, Amsterdam, the Netherlands; 3 Department of Neurosurgery, Amsterdam UMC, location VUmc, Amsterdam, the Netherlands

**Keywords:** Continuing professional development, motivation, medical specialists, self-determination theory, qualitative research

## Abstract

**Objectives:**

The aim was to investigate which factors influence the situational motivation of medical specialists and how situational and contextual motivation affect one another.

**Methods:**

A qualitative design was used, and a constructivist approach was adopted with the Self-Determination Theory of motivation as a framework. Twenty-two medical specialists from three medical centers in the Netherlands were recruited through convenience, snowball and purposive sampling and observed for two days each. At the end of the second observation day, a semi-structured interview was conducted. Data were transcribed and coded in an open manner. Themes were finalized through discussion and consensus.

**Results:**

Two-hundred and fifty hours of observation data together with the interview data identified that medical specialists experience six main themes influencing their situational motivation during a workday. Technical issues are influencing motivation negatively factors. Working with colleagues can be both a motivating factor and influence motivation negatively, e.g., filling in for each other through feelings of relatedness was motivating. Being in control of one’s own planning through feelings of autonomy was motivating. Patient care, especially in combination with teaching, stimulated specialists’ motivation.

**Conclusions:**

The results indicate that factors influencing motivation negatively are mainly tasks and organizational processes that distract from patient care or that compromise the quality of care. When optimizing the work environment of medical specialists, autonomous motivation and continuing professional development are stimulated.  These, in turn, can improve the quality of patient care and wellbeing of specialists.

## Introduction

In healthcare, the continuously changing work environment and societal demands, which require continuous adaptation during a workday, are an additional stressor to an already highly demanding work context.^1^^-^^4^ Although difficult to measure and quantify, previous research suggests that these developments and stressors may erode professionalism, influence the quality of care, patient satisfaction and increase the risk of preventable adverse events, job turnover or burn out among medical specialists.^5^^-^^10^ The latest studies in the Netherlands report that one in eight medical specialists experience symptoms that can be classified as burnout.^2^ According to the latest research into preventable adverse events (1035 per year) in hospitals in the Netherlands, knowledge and skills about technological developments and the resilience of the professionals need focus and improvement.^2^^,^^5^^,^^6^ Continuing professional development (CPD) is important as this can increase knowledge, skills, and resilience.^7^^-^^10^ Barriers for CPD have been identified, of which one is lack of motivation.^11^^-^^17^ Autonomous motivation stimulates performance, academic success and leads to positive wellbeing and greater resilience among medical students and other health care professionals.^11^^-^^17^ To engage physicians in CPD, appreciative inquiry into factors important for their motivation at work on a day to day basis (situational motivation) is necessary.^18^^-^^19^ However, there is a lack of literature, especially qualitative research, on the work motivation of medical specialists and on what motivates and demotivates them. Accordingly, the objective of this study is to investigate factors that influence the daily motivation for work of a medical specialist.

The literature on job satisfaction among physicians relates job dissatisfaction with an intention to leave, burn out and lower quality of patient care.^20^^-^^23^ However, the literature on the motivation of physicians is still scarce. Some literature on the motivation of health professionals is focused on a specific task, that is part of the job like teaching.^22^ Furthermore, most literature focusses on the motivation of health care workers in general (i.e. nurses, pharmacists, physicians) or/and by self-reported measurements like a survey.^20^^-^^23^ Using a more motivational focused framework will benefit our understanding of the mechanisms behind job satisfaction. A motivation focused framework that is broadly used and validated in many contexts (sports, education, parenting, health care) and across cultures, is the self-determination theory (SDT).^12^^-^^18^

SDT classifies two types of motivation, i.e. autonomous and controlled motivation.^12^^-^^18^ Autonomous motivation (AM) concerns intrinsic motivation (doing something out of interest or enjoyment), identified regulation (the appreciation of certain behavior as being personally valuable) and integrated regulation (personally endorsed values as part of oneself).^12^^-^^18^ On the contrary, controlled motivation (CM) implies that behavior is driven by the promise of reward or the threat of punishment (external regulation), or by internal pressure such as feelings of guilt or shame (introjected regulation). AM is considered to be the optimal type of motivation which leads to better performance, wellbeing and resilience. For a person to be autonomously motivated, three basic psychological needs have to be fulfilled. These needs are autonomy (experiencing a sense of volition), perceived competence (feeling capable of mastery at a task) and relatedness (feeling connected with peers).^18^^,^^24^^-^^26^ Moreover, there is a certain hierarchy in this construct in which different types of motivation exist at three levels of generality. Global, contextual and situational motivation refer to an individual’s motivation in (life in) general, in different life contexts (e.g. education, leisure, interpersonal relationships), and in a particular moment or situation respectively.^18^ In this study, situational motivation refers to each different task that a medical specialist has to conduct daily and the contextual motivation refers to their motivation for work or medical practice.

Little is known about the dynamic interplay between contextual and situational motivation. While past research has provided insight into the social and intrapersonal antecedents of motivation, the relationship between different levels of motivation lacks sufficient investigation.^18^^,^^24^^-^^26^ Repeatedly engaging in autonomously motivating activities at the situational level, together with experiencing their beneficial consequences plays a role in facilitating contextual autonomous motivation.^17^ The purpose of this research is to investigate which factors influence the situational motivation of medical specialists and how situational and contextual motivation affect one another. Accordingly, the following research questions have guided this study:

### Research Questions

·       Which factors in the work environment influence a medical specialist’s situational motivation?

·       How do these factors influence medical specialists’ motivation?

·       How does this situational motivation affect contextual motivation for medical practice?

We examined this by conducting a qualitative observational study. This adds to the field of motivation as there is a call to use qualitative methods instead of the current over-reliance on self-reported questionnaires.^27^ The global motivation was not taken into account as motivation at this level is quite stable. Knowing which factors influence a medical specialist’s motivation provides the opportunity to create the best possible environment for specialists to work in, which will positively influence their situational motivation and via this level also have a positive influence on his/her contextual motivation.^12^^,^^24^^-^^26^ This is expected to be beneficial to the delivered health care because when a doctor is motivated at the contextual level, it is more likely that this doctor has long-term motivation for medical practice and is motivated to invest in his professional development which leads to more wellbeing, resilience and better patientcare.^12^^,^^24^^-^^26^

## Methods

This is a qualitative study using observations, and interviews with an approach within the constructivist paradigm.^28^^-^^30^ In this paradigm, there is acceptance of reality and meaning as relative, produced through the interaction between the researcher and the researched, acknowledging the subjectivity of the researchers producing accounts of a social phenomenon.^28^^-^^30^

To get familiar with the population of medical specialists, to give better direction to the observation for this study, and to ensure that it is possible to get all the needed information from the observations for answering the research questions, we conducted a pilot study with observations.^31^ This pilot study yielded insight into which aspects could be observed and helped to decide on the focus and design for this observational study.^31^ To optimize data collection in this study, we adapted the design of the pilot study by adding a second day of observation, and we included medical specialists from both academic and peripheral hospitals.

### Setting

This study was conducted within three teaching hospitals in the Netherlands. These hospitals include a large academic teaching hospital, with patient care, education and research as core tasks, a large teaching hospital, with patient care, education as core tasks and to a lesser extent research task and an affiliated teaching hospital, with patient care and education as core tasks.

### Sample

The medical specialists were of different specialties and gathered by convenience, snowball and purposive sampling. Medical specialists were included until sufficiency was reached, i.e. sufficiency for gathering enough information to answer the research question.^28^^-^^30^ For transferability of findings and identification of common factors across disciplines different specialties were included (i.e., applying research findings from a particular study to a similar setting).^28^^,^^30^ In this study a medical specialist is a physician with a completed specialty training.

Ethical approval for this study was obtained from the local Institutional medical ethical Review Board. Written informed consent was obtained from all participants. However, informed consent was with minimal disclosure (offering generic rather than specific study information) to prevent participants from altering specific behaviors, which helps to minimize the observer effect in the study.

### Design

Direct observations were conducted by the first author through shadowing each participant for two days. Every participating specialist had the opportunity to choose two days in a row or at least within one week that suited him/her. This led to a variety of types of days. All specialists stated that the observation days were representative of typical workdays. The observation days started from the moment the medical specialist entered the hospital and ended when the specialist left the hospital at the end of the day. The focus of the observations was to unravel motivating factors and factors that influence motivation negatively during a workday. Therefore, SB observed what happened to the mood of a medical specialist -whether an event, activity, or situation was motivating or influencing motivation negatively. Differences in a mood of the medical specialist could be observed by smiles, active attitude, frowns or shaking heads when a specialist seemed happy, relaxed, irritated or stressed. Sometimes their mood could be told from statements that specialists made about being frustrated or motivating. Brief contemporary notes were taken during the observations, and extensive field notes were written up immediately after each daily observation.^28^^,^^30^ Besides field notes, the researcher kept a reflective diary to ensure a certain distance from the observation notes and to ensure the validity of the collected data.^28^^,^^30^ At the end of the observation day, participants were asked to draw a graph of their level of motivation during the day and talk about their thoughts on observed situations for stimulated recall and to ensure the trustworthiness of the gathered data. This served as member checking, and it strengthened the internal validity of the data because the observation could be discussed and viewed through the perspective of the participant. To understand how factors influence the motivation and to find the link between contextual and situational motivation, in-depth interviews were conducted at the end of day two of the observations.

### Analysis

The data from the observations and interviews were transcribed and coded concurrently with data gathering. Data were open coded in a constant comparative manner by two researchers SB and KN using qualitative analysis software Atlas.ti. To identify factors influencing motivation we used content analysis and to answer the second research question on how the factors influence specialists motivation another layer of content analysis with autonomy, competence and relatedness as sensitizing concepts was conducted. Differences in coding were discussed among the research team until agreed upon by all team members. In the final stage, the research team came to a consensus about the themes through selective coding and iterative discussion. This helped in establishing triangulation of data and validity of the findings.

### Researchers involved in this study

The research team of this study consists of one sociologist and four medical doctors of whom two are clinical specialists. The observer being a sociologist ensured the absence of inherent power dynamics in the relationship between the observer and the participants. Through her training as a sociologist, the first author could blend her perspective with those of the study participants and researchers involved in the collaborative analysis process.^28^^,^^30^ KN and SP are medical specialists and added the perspective of the healthcare work floor into this study. RK and GC are specialized and experienced in studies using SDT. In the spirit of reflexivity we acknowledge our assumption that motivation can change. We believe that a person can have different types or levels of motivation at certain points in time within a certain context. However, this assumption is theoretically supported.

## Results

In total, 22 medical specialists participated in this study, were observed for two days each and interviewed afterwards at the end of their second observation day. This resulted in approximately 20 hours of observation and around 35 minutes of audio interview per specialist. The medical specialists who engaged in this study were neurosurgeons, general surgeons, anesthesiologists, neurologists, ENT-surgeons, cardiologists, gynecologists, internists, pediatricians, gastroenterologists, ophthalmologists, psychiatrists, chest medicine physicians and emergency room specialists. Thirteen specialists were males, nine were female, and the average age was 46 (38-59) years. By observing and interviewing the medical specialists and thorough analysis of the qualitative data, different (de)motivating issues came up that we have classified into six themes: 1) technical issues, 2) working with colleagues, 3) patient care, 4) work environment, 5) organization, 6) other factors.

### Technical issues

All medical specialists complained and grumbled about issues concerning electronic patient files that they had to work with. This starts as irritating, but it becomes a factor that influences motivation negatively factor quickly when issues repeatedly appear during the day and continue to exist as a part of daily practice.

“This is so frustrating, everybody keeps complaining about these systems we have to work with, but they have decided not to organize a helpdesk, this makes for no nice atmosphere, of course, it is influences motivation negatively”. (Specialist no. 2)

When equipment for patient treatment in the outpatient department or at the operating theatre does not work properly, it becomes a factor that influences motivation negatively. The moment the specialist tries to adjust the microscope the way he wants it, he says:

“what a nonsensical case this thing  is, it never seems to work, setting this scope in the right position is always a fight and luckily I have some time now to do this.” (Specialist no. 4)

Medical specialists also raised issues regarding the different electronic patient file systems in other hospitals. First, these systems do not synchronize well with each other, and second, these take more time to work with than “old fashioned” pen and paper.

“This system is working so slow therefore we have two computer screens in the clinic so that I can fill out one question on the first screen and the next question on the second screen and so on. I would be much quicker if I just had a pen and paper. So, so frustrating!” (Specialist no. 17)

Specialists state that the systems in practice are neither aligned with their needs nor their way of thinking and working.

“Extremely clumsy…how this system was made. It is conceived at a distance, from behind a desk and not based on how we as doctors work with it”. (Specialist no. 13)

Some specialists had some concerns about the lack of ICT skills among the older population of specialists. All of these technical issues are experienced as influencing motivation negatively because they prevent medical specialists from doing their job properly and make it a frustrating experience.

### Working with colleagues

Medical specialists perceived working with colleagues both as motivating and influencing motivation negatively. Intercollegial contact is described as pleasant, fun and positive when colleagues can learn from each other and support each other. Another motivator is the fact that colleagues can share experiences and complement each other, creating a better healthcare outcome (inpatient care, for example).

“This gives me energy; nice and pleasant Intercollegial contact about patients in a good and positive way”. (Specialist no. 11)

Some specialists mentioned an informal consultation with colleagues as an example. Also being able to vent to colleagues about experiences, whether they are private or work-related, is a relief and an essential part of daily practice.

In contrast, the “inaccessibility” of colleagues and “stiff cooperation” with another department or a different hospital were mentioned as influencing motivation negatively. When the specialist calls with another hospital to obtain information about a patient, this hospital does not have a digital system for information letters so they cannot send it to him immediately. As the specialist hangs up the phone, he curses and says:

“really unbelievable, ridiculous that I am not able to receive this information about my patient”. (Specialist no. 20)

Also, colleagues who do not (want or dare to) consult each other and a different work mentality among colleagues are mentioned as influencing motivation negatively. Mainly because of these frictions, it feels like specialists are no longer working as a team to reach the same goal.

“The thing that demotivates is exactly the opposite of what makes working in a team with team spirit such a motivating factor, namely the feeling of not working together to reach the same goal for a patient or patient care in general”. (Specialist no. 1)

### Patient care

Patient care is another factor that is experienced both as motivating and influencing motivation negatively. Satisfied patients, good patient outcomes and innovative operations are examples of when patient care is experienced as motivating.

“My heart is inpatient care.” (Specialist no. 1)

“If I have patient contact, then it does not matter if I have to work 60 hours a week.” (Specialist no. 8)

Patient care in itself is motivating as specialists state that they like the challenge of the different cases that they see and get to treat every day. Adjectives that came up during the interviews and observations about patient care were: humorous, relaxed, energetic, efficient and effective, and nice or fun. For medical specialists, patient care becomes even more motivating when it is combined with teaching.

“Making people better by taking care of my patients and transferring my knowledge to the next generation so that they can hopefully become even better than I am, is what I love the most about this job.” (Specialist no. 6)

Patient care influences motivation negatively when “bad patientcare” is provided, when having a patient with hazy or unsolvable complaints or when a patient is demanding.

“Consultation hour from hell, all people with vague complaints that you cannot do much about and on top of that it is extremely busy. Normally I really like my consultation hours, but this was just too many hazy and unsolvable complaints altogether.” (Specialist no. 11)

Specialists did not feel like they were able to live up to the wishes of these patients or it took away time that they could have spent on “successful patient care". Medical specialists also experienced that a language barrier between a specialist and a patient can create difficulties. This can be influencing motivation negatively when there is not enough mutual understanding because it can hamper a specialist in providing proper patient care.

When it is challenging to communicate with a patient, the medical specialist gets irritated, I hear some sighing, see eyebrows go up, and the specialist is restless behind his desk. Afterwards, the specialist says:

“communication was difficult because this patient did not speak Dutch and only moderately to no English.” (Specialist no. 9)

On the contrary, some specialists see a language barrier as a challenge and therefore as motivating.

### Time pressure

Time pressure is a factor that is influencing motivation negatively and mentioned the most. Time pressure affects all tasks of medical specialists on a daily basis, but most of all patient care. Another factor that is related to time and experienced as influencing motivation negatively is: not having the autonomy over one’s own agenda. It actually gives the specialists headaches.

“Alright, two new patients at the same time in the schedule, so there is no chance that this will work out and I will run out of time with this schedule. But when I just asked the assistants at the reception desk how this is possible, they answered that they don’t know. That really gives me an instant headache because they are there, exactly, to make sure this does not happen.” (Specialist no. 4)

Also, the amount of administration including email that takes away time from patient care is expressed as influencing motivation negatively and a “frustrating burden”.

“email is also some kind of a disaster.” (Specialist no. 7)

### Work environment

Medical specialists commented that in a hospital, little attention is paid to workspaces and facilities. Most specialists share their workspace with too many colleagues. The specialists in the office discuss on the division of the rooms in clinic today:

“one room has a window, the other one is dark, small and has fluorescent lighting, so it is nice to switch rooms halfway through the day for some more work enjoyment.” (Specialist no. 19)

They also mention poor housing and cleaning and only a few or no possibilities for having lunch or meetings outside and some leisure activities (like having a gym space to work out during breaks or nightshift or decent sleep facilities for the ones on a nightshift) around the hospital.

“When I see this sign with the name of the hospital, and I see that it is yellowed with age or from dirtiness, along with dust on the floor or nasty windows when I walk into the hospital, I just feel ashamed actually.” (Specialist no. 1)

“There are some rooms downstairs that have a bed where you can sleep and a chair. It would be nice to have a shower there as well so that you can freshen up a bit when spending 24 hours or longer in this hospital.” (Specialist no. 10)

### Organization

Looking at the organization of their hospital, specialists experience a lack of personnel and a shortage of resources, especially the ones necessary for patient care. As a consequence, the specialists mention a limited possibility for the right patient care at times.

“Practice is conforming to the regulations instead of the regulations conforming to practice. In addition, interventions are experienced as being implemented very slowly.” (Specialist no. 15)

Specialists also state that “the system is taking away from motivation because it is already set up on having long working days and even patients can see and also mention that they notice that doctors are so busy and do not have enough time for their job.” Other factors that influence motivation negatively are the imposed management tasks and other extra tasks or activities.

When the specialist is at his desk doing some management tasks, he says:

“This really drives me crazy, I actually do not care about this, nor am I trained for this.” (Specialist no. 8)

Specialists mention that they experience a gap between training and practice. They say that this gap creates wrong expectations.

### Other factors

Some final issues that influence motivation negatively that came up are financial issues/ budget cuts that specialists have to deal with. Most frustrating are the ones that influence patient care and also the discussions that specialists need to have with their patients during consultation.

“The discussion of (too) expensive medicines is unfortunately increasingly a topic of conversation in the doctor’s office. It really makes me grumpy, because I want to help people as well as I can and do not want to concern myself with this.” (Specialists no. 23)

Finally, specialists mention the difficulty of the need to always be available (mostly on the phone).

“These phones are always interrupting me in everything, you should have an empty schedule when it is your turn to take the phone with you. I cannot even work for three minutes straight without being disturbed, if you really want to work in concentration, well there is just no chance.” (Specialist no. 5)

### Effect on contextual motivation

As described above, some factors start out as just irritating but develop or continue to exist, and turn out to be influencing motivation negatively. The temporary diminishing of motivation in a particular situation is surmountable if there is a good quality of contextual motivation. The contextual motivation will make up for the diminishing situational motivation.

“Of course, there are some issues that cost a lot of energy and are completely frustrating and no fun. But if it only takes a short amount of time, then that is not a problem I am concerned about or will raise my voice for”. (Specialist no. 15)

However, when different factors persist on a daily basis, this can lead to more structural diminishing of motivation and influence the contextual motivation negatively. For example, the housing issues that continue to exist make specialists feel unequal:

“Some specialties have beautiful office spaces as other specialties are just put in an overcrowded room together, and the reason provided is: that we only have to see patients all day and therefore do not need an office. Well, I guess all specialists have to see patients all day right?!” (Specialist no. 3)

Also technical issues can start out as irritating but become factors that influence motivation negatively because it is a frustrating factor on a daily basis. No solutions are explored, and the negative consequences for multiple factors at the same time are experienced several times a day. If the situational motivation of medical specialists keeps getting diminished by these issues, they might not be surmountable anymore. This is when the good quality of contextual motivation for work (the autonomous contextual motivation for work) is affected negatively by a diminishing situational motivation. When the quality of the contextual motivation diminishes, the motivation for medical practice suffers and with it the wellbeing, resilience and performance of the medical specialists.

Therefore, it is necessary to sustain the quality of medical specialists contextual work motivation. To stimulate a specialist’s contextual motivation, the situational work motivation needs to improve. Repeatedly engaging in motivating work activities at the situational level, together with experiencing their beneficial consequences, plays a role in facilitating contextual autonomous work motivation.

## Discussion

This study aimed to determine the motivating and influencing motivation negatively factors for medical specialists in their daily work. A second aim was to see how these factors influence the situational and contextual motivation of medical specialists.

We found six themes of motivating factors and factors that influence motivation negatively. The themes were technical issues, working with colleagues, patient care, work environment, organization and other factors. These are in line with factors found before among a small group of Dutch medical specialists and among residents.^31^^,^^40^^,^^41^ Patient care and working with colleagues were found to be both motivating and influencing motivation negatively in the present study. Patient care in itself is experienced as motivating because of the challenge of facing different cases every day and because good patient outcomes are satisfying. In earlier studies patient care was found to be most motivating for medical specialists as it combines the fulfillment of all three basic psychological needs of SDT.^24^^,^^26^^,^^31^ Autonomy because the specialist can decide on the treatment; competence because the specialist is able to provide the best care according to him/her; and relatedness, because the specialist (can) feel(s) related to the patient.^24^^,^^26^^,^^31^ On the contrary, bad patient outcomes, patients with issues that cannot be solved or when demanding

patients are especially influencing motivation negatively because it takes away from the fulfilment of the need for competence.^24^^,^^26^^,^^31^         

Working with colleagues is found to be motivating when there is smooth collaboration and when specialists learn from each other or complement each other’s expertise, and achieve better results. Working with colleagues, in a manner that is experienced as positive, is motivating because it fulfils the need of relatedness among medical specialists.^24^^,^^25^^,^^31^ Working with colleagues is influencing motivation negatively when it leaches from being related to one another.^24^^,^^25^^,^^31^ In line with these results specialists in the present study experienced that this led to the feeling that they were not working as a team towards a common goal.

Technical issues in both information technology and equipment in the operating theatre or during the consultation is another factor that influences motivation negatively that was brought up frequently. Medical specialists mainly mentioned that the electronic systems that they have to work with are not aligned with their needs in practice and just produce delays instead of facilitation. Specialists raised concerns about the electronic system and lack of technical skills to work with new software, as it makes them feel incapable of providing the best treatment. This finding is in line with results from earlier research, in which technical issues were found to be amongst the most influencing motivation negatively factors and can decrease feelings of competence.^31^

Work environment and organization are two other themes of influencing motivation negatively factors. Specialists mentioned the healthcare system as a whole as this is set up in a way that already makes for long working days by having fixed times during a day for obligatory meetings. It also makes time pressure inevitable by having, for example, only ten minutes to see a patient in the clinic. Administrative tasks and extra management tasks are experienced as influencing motivation negatively because specialists are not trained for these, nor feel competent in carrying them out. Decreased feelings of competence lead to poorer quality of motivation, according to the SDT.^11^^,^^12^ The little attention that is paid towards the accommodation of the hospital and housekeeping is irritating for specialists too. Especially when it comes to having some comfort and possibilities to relax or rather de-stress at the hospital because it provides the opportunity to relax a bit during or after a day of hard work or between shifts. Being able to relax and get the adrenaline or stress back to a low point is necessary because when stress levels remain high for a long time, this can lead to chronic stress and even burnout.^1^^,^^5^^,^^6^

Having to deal with the above-mentioned issues in a work environment that is already busy and high demanding in itself, can provide extraneous cognitive load. Especially since these issues are experienced simultaneously. Cognitive load refers to the way information or tasks are presented and in turn being processed in the working memory.^35^^,^^36^ Extraneous cognitive load typically creates error or some kind of interference in the task at hand, which might happen if too many tasks need to be processed simultaneously or the cognitive capacity reaches its limits.^35^^,^^36^ Hence, another reason why colleagues should communicate and work well together and why possibilities to relax are important.

According to SDT, repeatedly engaging in autonomously motivating activities (at the situational level), together with experiencing their beneficial consequences, will play a role in facilitating contextual autonomous motivation.^12^^,^^37^ This might also work the other way around. Repeatedly dealing with frustration or irritation at the situational level can play a role in decreasing contextual motivation. Therefore, this study aimed to see how these factors influence not only situational motivation but also contextual motivation. Medical specialists state that factors or issues can start out as frustrating or irritating, but if they continue to exist, they become influencing motivation negatively. Especially the issues that the specialists have to deal with daily. When these issues are short and/or temporarily of nature, the diminishing motivation in that particular situation is probably surmountable because specialists still have a contextual motivation that is of good quality. Persistent feelings of frustration, demotivation and inequality can negatively influence the contextual motivation of specialists. This, in turn, can decrease specialists wellbeing, performance and thus the quality of patient care.

### Implications and future research

All factors in this study that influence motivation negatively are seemingly regular or simple factors but do evoke emotions, feelings of frustration and demotivation, and sometimes physical distress (headaches) among medical specialists. If the work environment supports motivation for medical specialists this might be a way of improving the wellbeing among the health professionals (i.e. burnout rate) or the preventable adverse events. Eliminating these factors that influence motivation negatively in the work environment by applying simple solutions, can have a great effect on medical specialist’s motivation, wellbeing and quality of work. Some literature suggests that changing people’s position and/or the color, lighting or temperature in a meeting room can have great positive effects on the productivity and the ethos or ambiance.^38^^,^^39^ There might be some fairly simple solutions for the factors in this study as well.  Administrative tasks, for example, can be done by an assistant, e.g. ascribe that works along with a medical specialist when seeing patients. For technical issues, it would be helpful to have a helpdesk available at all times. In addition, make sure to teach all specialists how to work with new software and systems when these are introduced and involve specialists in designing new technical systems as they are the ones that are going to use them and know what is needed. Creating a culture at the workplace where specialists can openly discuss their frustrations, experiences, and address others’ behaviors and attitudes when necessary, might help in getting a more positive relationship between colleagues and in turn more positive cooperation possibilities. Finally, more attention should be paid in providing medical specialists with the right equipment and (clean) housing to do their jobs properly.

For future research, it would be interesting to see which simple solutions work best, are appreciated by the specialists and are easy to implement. However, even with solutions for a lot of factors, some will remain, and others will come up.  Therefore it is interesting to find out how medical specialists cope with the issues that demotivate them.

### Strength and limitations

More motivated specialists or specialists interested in the subject of motivation and professional development could have been more drawn to participate in this study. Or because we have used snowball sampling, the participants may have identified people who feel the same way they do, therefore not providing us with an adequate and representative sample. This could give a more positive image of the motivation of medical specialists. However, if we indeed have more motivated specialists in our sample, and still found these influencing motivation negatively factors and barriers for medical specialists to do their work at an optimal level. These factors seem to be of great importance for medical specialist’s motivation and might have even greater impact on less motivated specialists. The study is conducted in the context of a specific environment and country and involved the participation of only a selected number and types of specialties. Therefore, the findings may not be applicable to other clinical practices and/or other specialties, however, qualitative research is about transferability and not generalizability.

## Conclusions

Technical issues, working with colleagues, patient care, work environment, organizational and other factors are found to be the most motivating or influencing motivation negatively for medical specialists during their daily work. The results indicate that factors influencing motivation negatively are mainly tasks and organizational processes that distract from patient care or that compromise the quality of care. A work environment for medical specialists that includes sufficient time for patient care and transferring knowledge (teaching) optimizes motivation in daily work. By optimizing the work environment of medical specialists, quality of patient care and wellbeing of specialists can improve.

### Acknowledgements

The authors would like to thank all medical specialists who participated in this study.

### Conflict of Interest

The authors declare that they have no conflict of interest.
